# Dabigatran‐induced esophagitis with full circumferential blue pigmentation

**DOI:** 10.1002/deo2.271

**Published:** 2023-07-17

**Authors:** Tomonori Araki, Kohei Hayashi, Yuki Sonoda, Takuya Honda, Yoshifumi Imamura, Yuji Koide, Hisayuki Hamada, Kazuhiko Nakao

**Affiliations:** ^1^ Department of Gastroenterology and Hepatology Graduate School of Biomedical Sciences, Nagasaki University Nagasaki Japan; ^2^ Medical Education Development Center Nagasaki University Hospital Nagasaki Japan; ^3^ Nagasaki Memorial Hospital Nagasaki Japan

**Keywords:** atrial fibrillation, dabigatran, drug‐related side effects and adverse reactions, esophagitis, pigmentation disorders

## Abstract

Dabigatran is a useful and widely used drug for stroke prevention in patients with atrial fibrillation. However, it has been reported to cause esophagitis. Herein, we report the case of a 77‐year‐old man with dabigatran‐induced esophagitis with blue pigmentation, which is known to be a rare adverse effect. The patient presented to our hospital with a tightness of the chest and anorexia. Computed tomography revealed a thickening of the entire esophageal wall, with an upper esophageal predominance. Esophagogastroduodenoscopy was performed, which showed that the cervical and upper thoracic esophagus had blue pigmentation with edematous changes, partial narrowing, and longitudinal sloping. We replaced dabigatran with edoxaban, a similar anticoagulation medication. The patient was closely monitored for 1 month after switching to edoxaban. The follow‐up esophagogastroduodenoscopy showed marked improvements, revealing resolution of the bluish discoloration and edematous changes, and the patient's complaints regarding the tightness of the chest and anorexia were also resolved. It is important to recognize that such side effects can occur with dabigatran, a drug that is frequently used in daily practice. Considering the fact that strong edematous changes can cause indigo carmine pigmentation associated with dabigatran stagnation, we recommend switching to another anticoagulant if esophagitis occurs during dabigatran administration.

## INTRODUCTION

Atrial fibrillation (AF) is a common heart condition characterized by an irregular heartbeat that can increase the risk of stroke.^1^ Anticoagulants are commonly prescribed for stroke prevention in patients with AF, and direct‐acting oral anticoagulants have become an increasingly popular alternative to traditional anticoagulants, such as warfarin. Dabigatran is a direct‐acting oral anticoagulants that has been approved for stroke prevention in patients with AF and is known for its predictable pharmacokinetics, rapid onset, and twice‐daily dosing without the need for monitoring.^2^

Esophagitis is a known adverse effect of dabigatran^3^, ^4^, ^5^, ^6^, ^7^, ^8^; however, there are only a few reports of the development of esophagitis with blue pigmentation.^4^ Herein, we present a case of dabigatran‐induced esophagitis with blue pigmentation, which is a rare adverse effect reported in the literature. This report aims to emphasize the importance of considering this rare adverse effect of dabigatran and provide insight into the management of this condition.

## CASE REPORT

**FIGURE 1:**
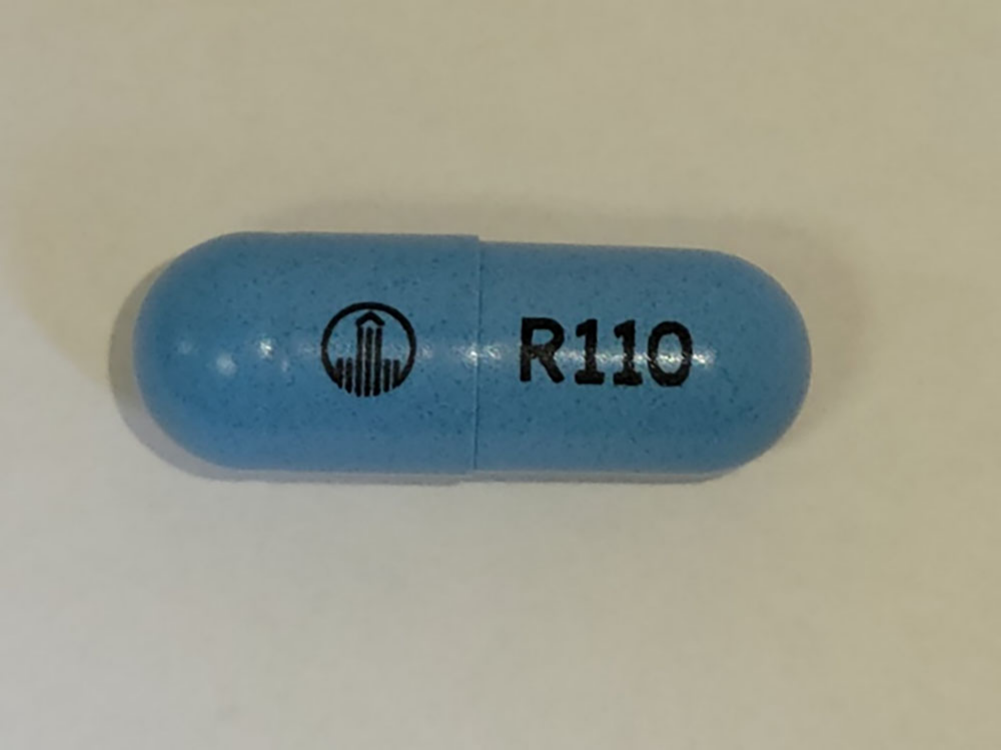
Dabigatran 110 mg capsule contains indigo carmine.

**FIGURE 2:**
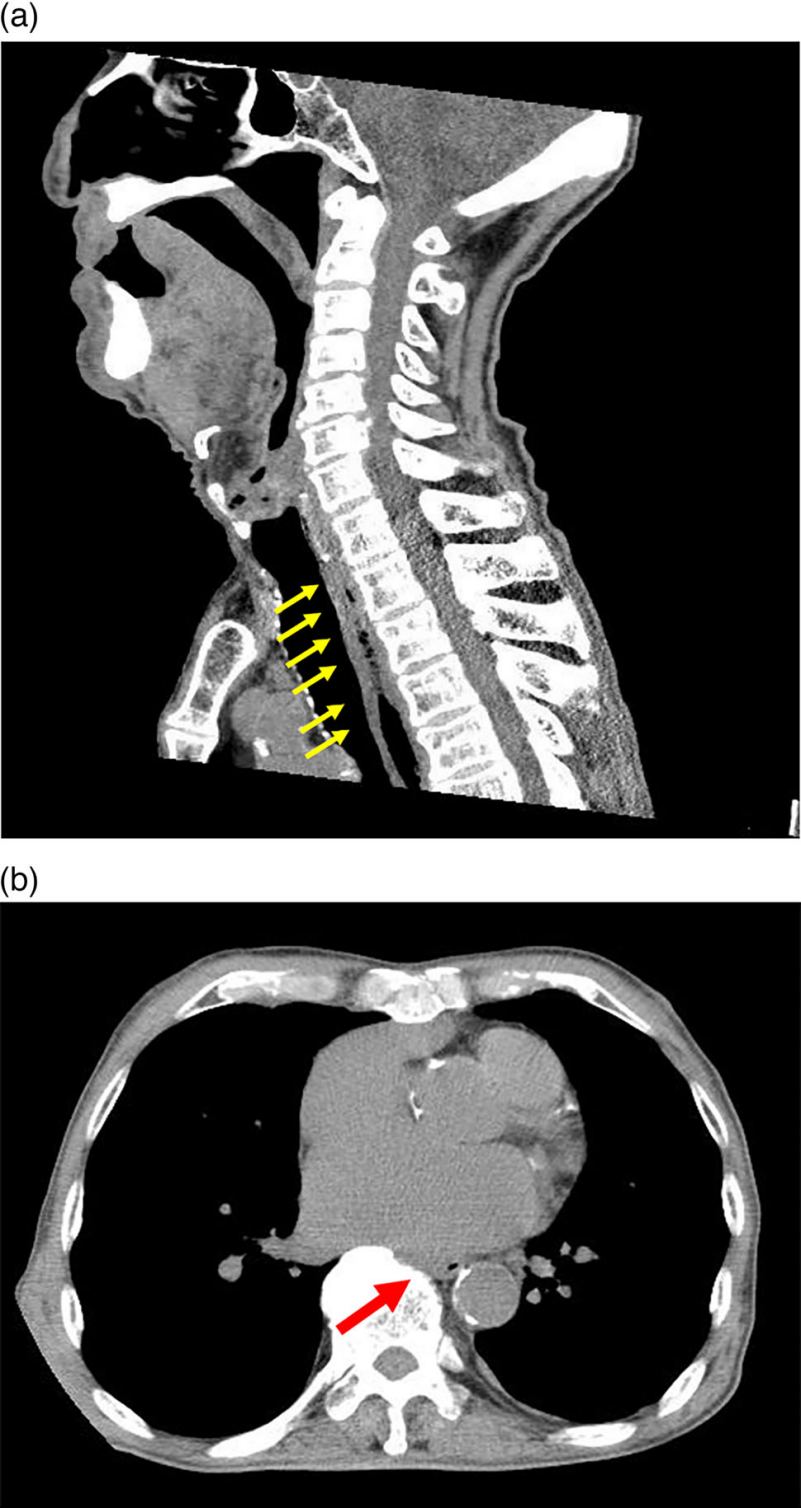
Sagittal computed tomography reveals thickening of the entire esophageal wall with an upper esophageal predominance (a). Horizontal computed tomography shows compression of the esophagus by the left atrium (b).

**FIGURE 3:**
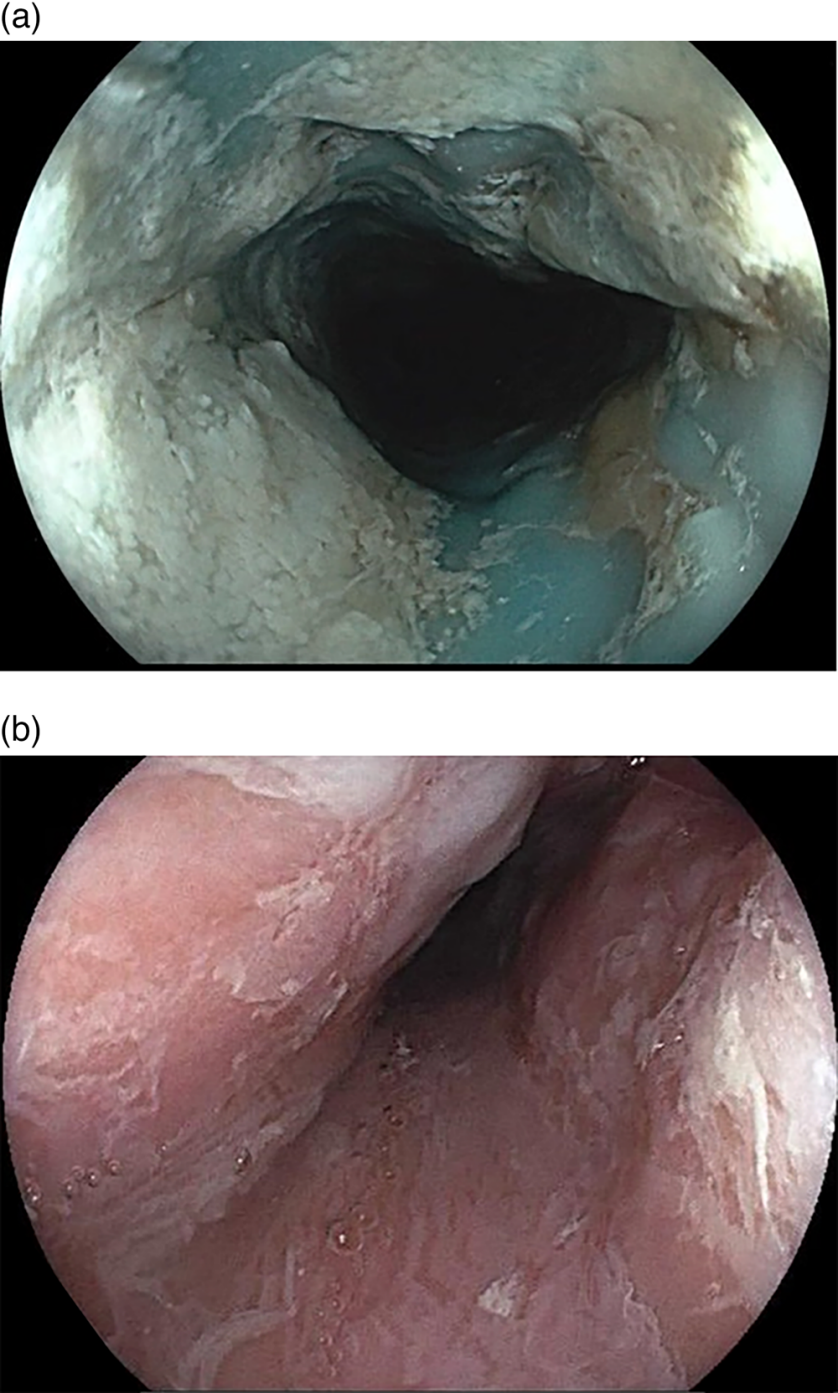
The cervical and upper thoracic esophagus show blue pigmentation, edematous changes, partial narrowing, and longitudinal sloughing of esophageal casts (a). Edematous changes, partial narrowing, and longitudinal sloughing of esophageal casts are observed throughout the esophagus (b)

A 77‐year‐old man with a tightness of the chest and anorexia presented at our institution. He received dabigatran 110 mg twice daily for 5 years for non‐valvular AF (Figure 1). Upon examination, computed tomography revealed a thickening of the entire esophageal wall, with an upper esophageal predominance (Figure 2a,b). Esophagogastroduodenoscopy was performed, which revealed that the cervical and upper thoracic esophagus had blue pigmentation (Figure 3a). Further, edematous changes, partial narrowing, and longitudinal sloughing of esophageal casts were observed throughout the esophagus (Figure 3a,b).

Blood tests showed an elevated white blood cell count of 14,030/μl; however, the serum concentration of C‐reactive protein was within the normal range. No anemia was observed, and the liver function, renal function, and electrolyte levels were normal. Creatine kinase levels were within the normal range, with no findings suggestive of bleeding, necrosis, or strangulation.

**FIGURE 4:**
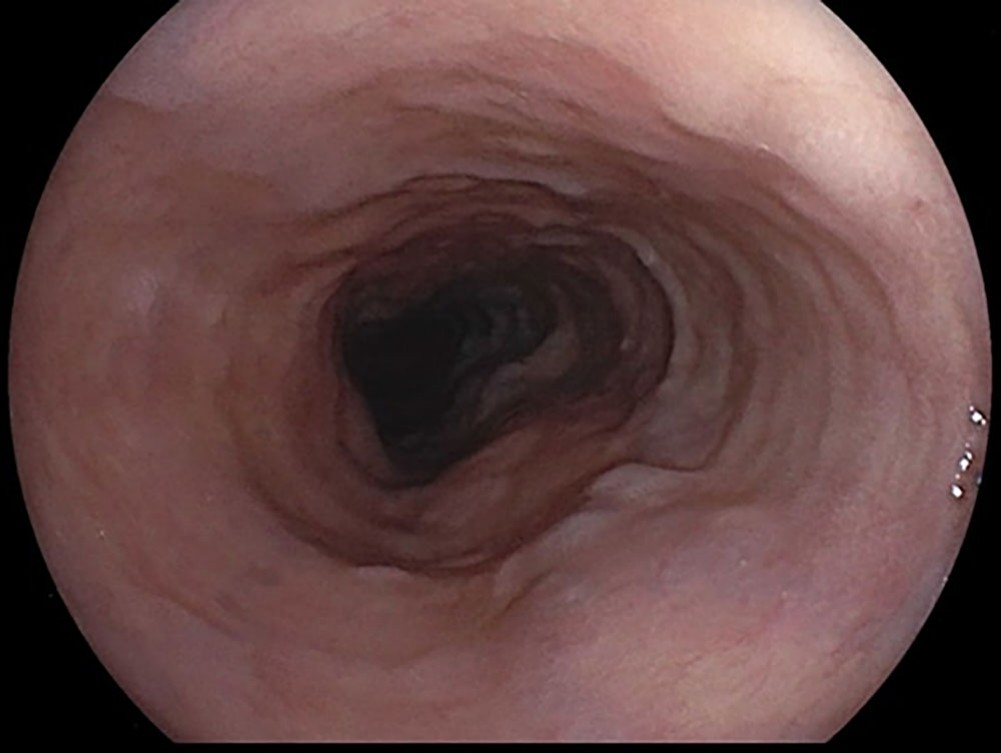
One month after the switch to edoxaban, esophagogastroduodenoscopy shows a significant improvement, revealing the resolution of the bluish discoloration and edematous changes.

The edematous changes were a manifestation of esophagitis. Since no other drug, the patient was taking had a blue appearance or contents, which included bisoprolol fumarate, famotidine, clotiazepam, cilnidipine, azilsartan, and ramelteon, we considered that the dabigatran tablets had lodged into the esophagus, and the esophagus was stained with indigo carmine resulting in the blue pigmentation. In addition, dabigatran is known to have an esophagitis‐prone profile,^4^ containing tartaric acid, a large drug with a long diameter of approximately 20 mm; therefore, we determined that changing the drug would be effective. Subsequently, we replaced dabigatran with edoxaban, a similar anticoagulant. The patient was closely monitored for 1 month after switching to edoxaban. The follow‐up esophagogastroduodenoscopy showed significant improvements, revealing the resolution of the bluish discoloration and edematous changes (Figure 4), and the patient's complaints regarding the tightness of the chest and anorexia were also resolved.

## DISCUSSION

In this case report, we presented a case of a 77‐year‐old man who developed dabigatran‐induced esophagitis with blue pigmentation. Dabigatran is a direct thrombin inhibitor commonly used for stroke prevention in patients with AF.^2^ It has a fixed‐dose scheme and does not require monitoring, making it an attractive option for many patients. However, our case report highlights a rare adverse effect of this medication that may be potentially serious because this condition is characterized by inflammation and discoloration of the esophagus, leading to difficulty in swallowing and pain.

Esophagitis is a well‐known adverse effect of many medications, including non‐steroidal anti‐inflammatory drugs and bisphosphonates.^8^ Although there have been several reports of dabigatran‐induced esophagitis,^3^, ^4^, ^5^, ^6^, ^7^, ^8^ only a few reports have documented bluish discoloration of the esophagus. Additionally, longitudinal sloughing of esophageal casts is common in dabigatran‐induced esophagitis. In our case report, the patient developed blue pigmentation, which was previously reported as a rare adverse event,^4^ in addition to the typical symptoms of esophagitis.

In the present case, as in a previous report, esophagitis with keratinization was observed. The patient developed edematous changes, which could be observed on computed tomography, and also complained of a tightness in the chest. Therefore, we believed that the oral medication passed through the esophagus for a longer time than usual, during which indigo carmine, the dye in dabigatran tablets, may have pigmented the esophagus. As no other concomitant oral drug had a coloration or content as blue as indigo carmine, we assumed that dabigatran tablets were the cause of discoloration.

Post‐diagnosis, the symptoms gradually improved over the next month, highlighting the importance of early recognition and prompt management of this adverse effect.

The selection of edoxaban as an alternative anticoagulant was based on its different mechanism of action and lower risk of esophagitis.^9^ However, it is important to note that individual patient factors and medication interactions must be considered when choosing an alternative anticoagulant.

In a previous report regarding dabigatran‐induced esophagitis, proton pump inhibitors (PPIs) were administered before the onset of the disease in approximately half the patients included; thus, PPIs are not considered useful for this condition, and no additional PPIs were administered in this case.^3^ In patients receiving dabigatran, the use of histamine‐2 receptor‐antagonists (H2RAs), PPIs, cyclooxygenase‐2 inhibitors, and nonselective non‐steroidal anti‐inflammatory drugs; age 75 years or older; and female sex and non‐Caucasian ethnicity were reported as risk factors for upper gastrointestinal adverse events.^10^ Our patient was treated with H2RAs, aged over 75 years old, and non‐Caucasian. However, these are risk factors for upper gastrointestinal adverse events, and it is not clear whether they are risk factors for dabigatran‐induced esophagitis. Dabigatran is available in 75 and 110 mg formulations, and the risk of developing upper gastrointestinal adverse events is the same for both^10^; however, indigo carmine is used only in the 110 mg formulation.

Our case findings were not in line with those of previous reports. In the present case, esophagitis was predominantly observed in the cervical and upper esophagus, whereas previous reports have focused on the middle and lower esophagus. Dabigatran‐associated esophagitis usually develops within 2 years after the start of dabigatran administration; however, this case occurred 5 years after the start of dabigatran administration.

Although the only organic abnormality of the esophagus was mild esophageal hiatal hernia, cardiac enlargement with left atrial enlargement was observed, which worsened over time. Therefore, it is possible that dabigatran gradually increased the time of arrest in the esophagus with the left atrial enlargement over a prolonged period.

We suspected that edema without pigmentation in the lower esophagus occurred first as in other cases of dabigatran‐induced esophagitis, and because the tablets were large (approximately 20 mm),^4^ they probably remained in the upper esophagus for a long time, resulting in prolonged exposure to dabigatran on the upper esophagus of the edematous area.

In addition, the lesions may have been more concentrated in the neck and upper esophagus because the patient was lying down for longer periods and not drinking enough water, both of which are important to avoid during dabigatran administration.^4^

In addition, it is possible that the effects of dabigatran on the esophagus may have accumulated due to prolonged oral administration. To prevent these problems, it is important to provide medication instructions, such as avoiding lying down shortly after taking the medication, drinking enough water when taking the medication, and taking the medication between meals.^4^

In conclusion, this case report highlights the importance of recognizing the side effects that can occur with the commonly used dabigatran and considering alternative anticoagulant options in such patients. Further research is needed to better understand the pathophysiology of this side effect and develop effective prevention and management strategies.

## CONFLICT OF INTEREST STATEMENT

None.
